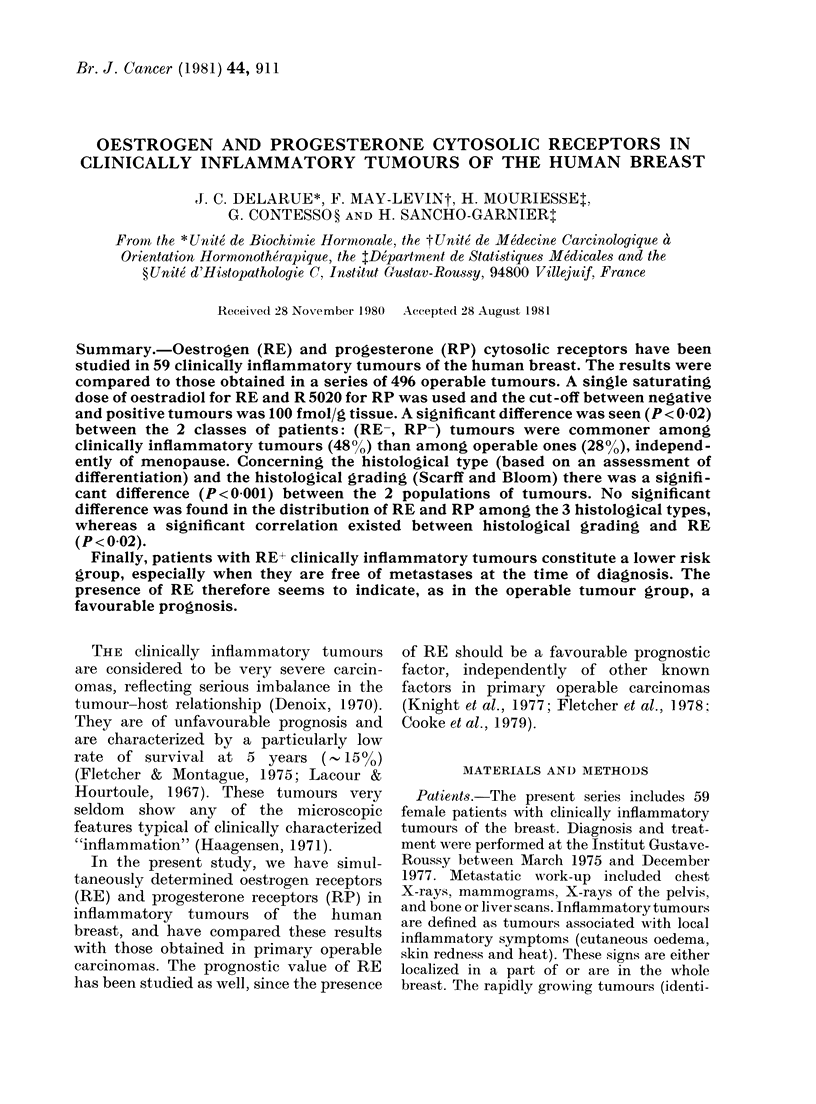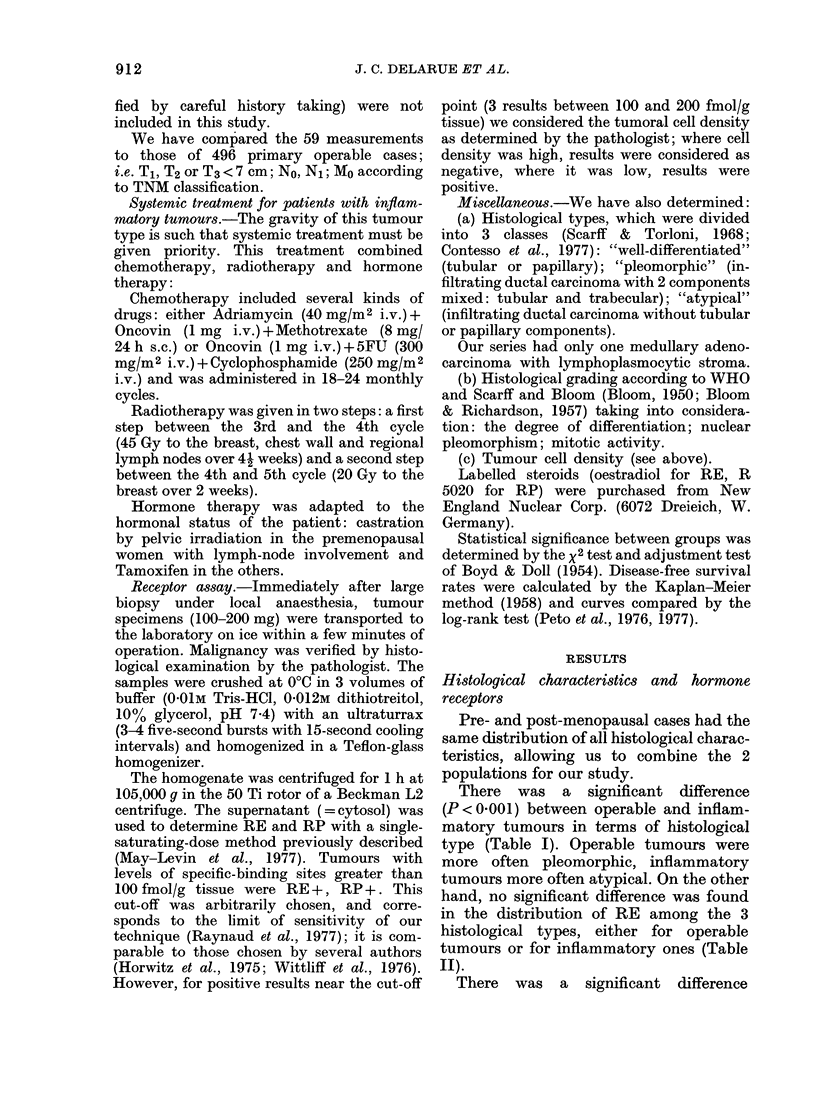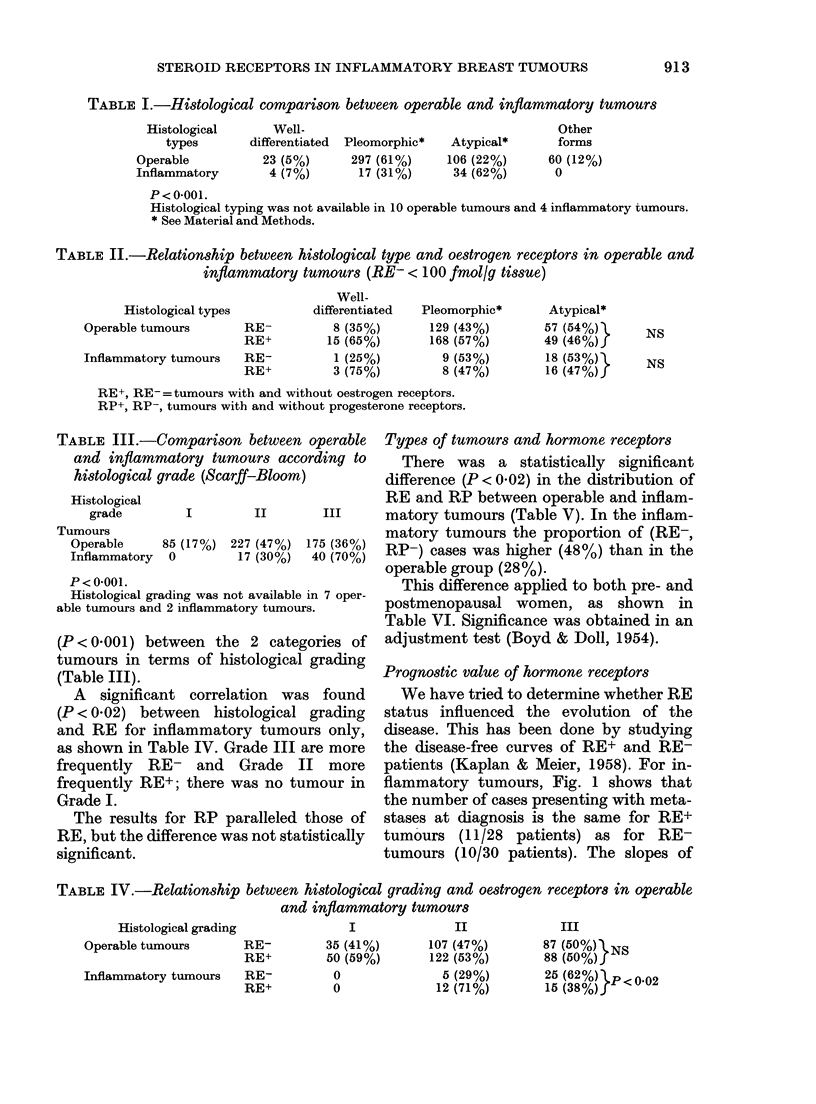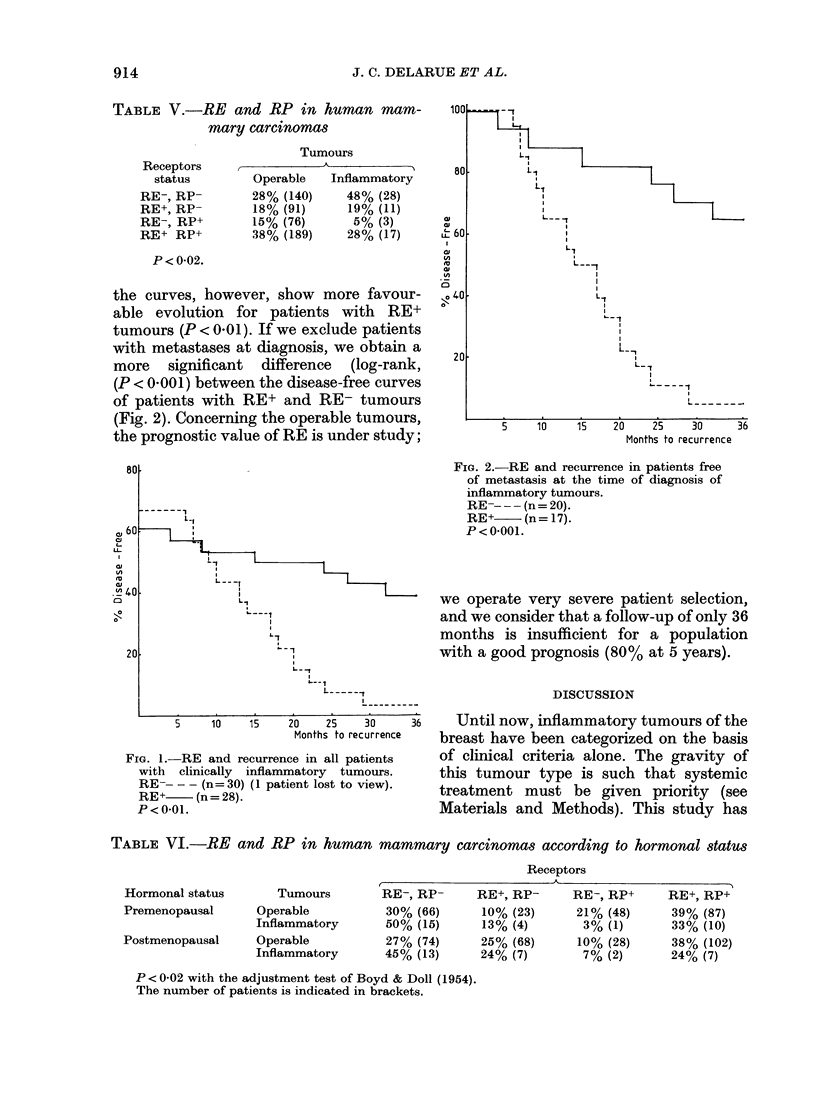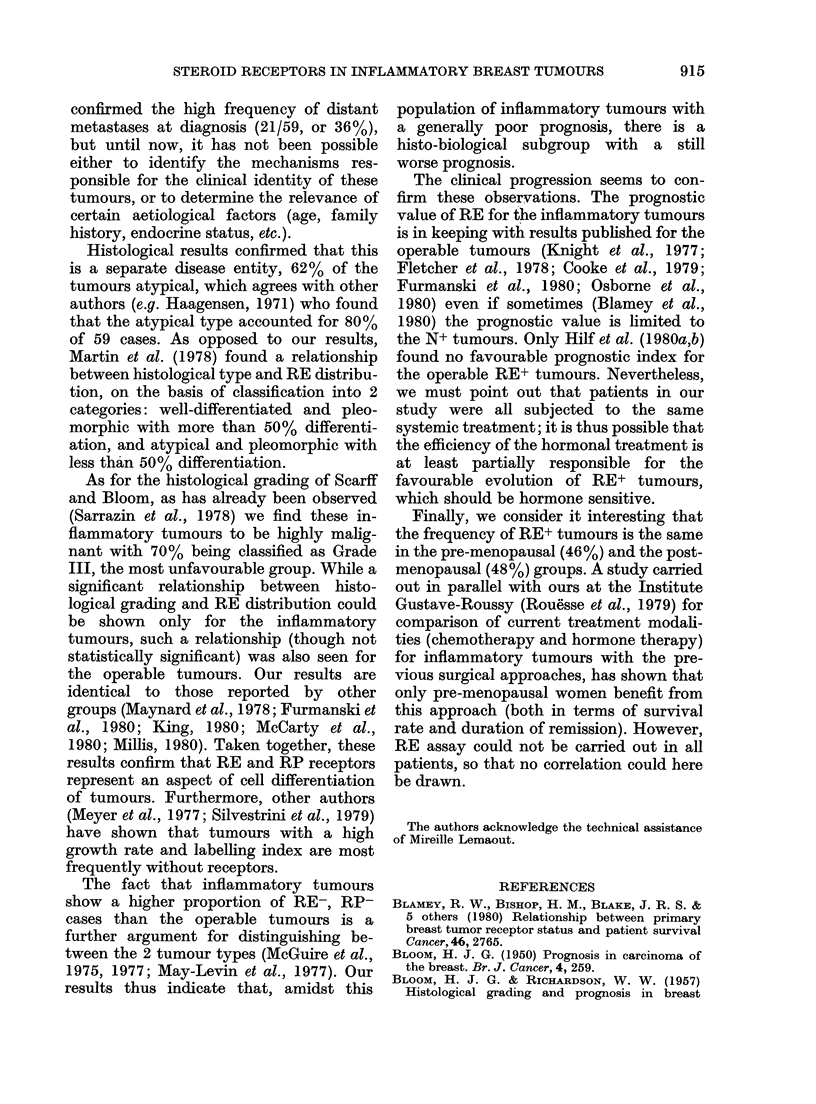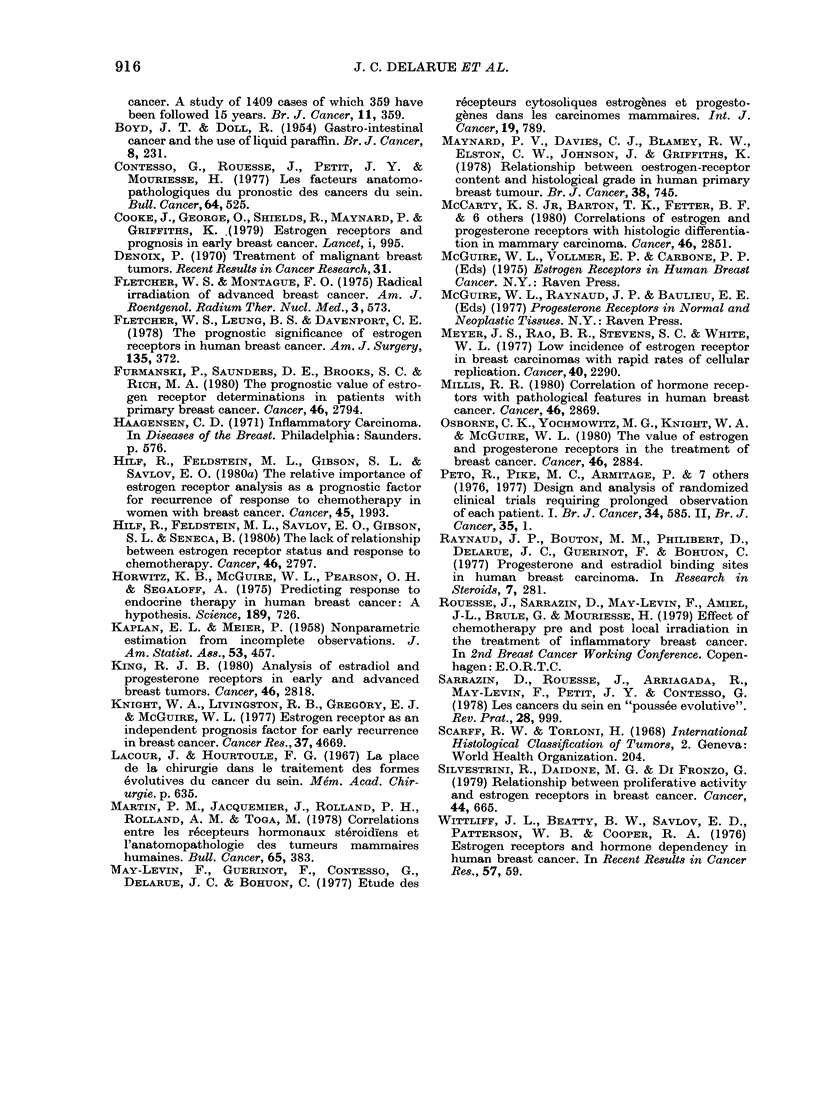# Oestrogen and progesterone cytosolic receptors in clinically inflammatory tumours of the human breast.

**DOI:** 10.1038/bjc.1981.291

**Published:** 1981-12

**Authors:** J. C. Delarue, F. May-Levin, H. Mouriesse, G. Contesso, H. Sancho-Garnier

## Abstract

Oestrogen (RE) and progesterone (RP) cytosolic receptors have been studied in 59 clinically inflammatory tumours of the human breast. The results were compared to those obtained in a series of 496 operable tumours. A single saturating dose of oestradiol for RE and R 5020 for RP was used and the cut-off between negative and positive tumours was 100 fmol/g tissue. A significant difference was seen (P less than 0.02) between the 2 classes of patients: (RE-, RP-) tumours were commoner among clinically inflammatory tumours (48%) than among operable ones (28%), independently of menopause. Concerning the histological type (based on an assessment of differentiation) and the histological grading (Scarff and Bloom) there was a significant difference (P less than 0.001) between the 2 populations of tumours. No significant difference was found in the distribution of RE and RP among the 3 histological types, whereas a significant correlation existed between histological grading and RE (P less than 0.02). Finally, patients with RE+ clinically inflammatory tumours constitute a lower risk group, especially when they are free of metastases at the time of diagnosis. The presence of RE therefore seems to indicate, as in the operable tumour group, a favourable prognosis.


*Br. J. Cancer* (1981) **44**, 911

### OESTROGEN AND PROGESTERONE CYTOSOLIC RECEPTORS IN CLINICALLY INFLAMMATORY TUMOURS OF THE HUMAN BREAST

J. C. DELARUE*, F. MAY-LEVIN†, H. MOURIESSE‡, G. CONTESSO§ AND H. SANCHO-GARNIER‡

*From the *Unité de Biochimie Hormonale, the †Unité de Médecine Carcinologique à Orientation Hormonothérapique, the ‡Départment de Statistiques Médicales and the §Unité d'Histopathologie C, Institut Gustav-Roussy,* 94800 *Villejuif, France*

Received 28 November 1980 Accepted 28 August 1981

**Summary.—Oestrogen (RE) and progesterone (RP) cytosolic receptors have been studied in 59 clinically inflammatory tumours of the human breast. The results were compared to those obtained in a series of 496 operable tumours. A single saturating dose of oestradiol for RE and R 5020 for RP was used and the cut-off between negative and positive tumours was 100 fmol/g tissue. A significant difference was seen ($P<0{\cdot}02$) between the 2 classes of patients: ($RE^-$, $RP^-$) tumours were commoner among clinically inflammatory tumours (48%) than among operable ones (28%), independently of menopause. Concerning the histological type (based on an assessment of differentiation) and the histological grading (Scarff and Bloom) there was a significant difference ($P<0{\cdot}001$) between the 2 populations of tumours. No significant difference was found in the distribution of RE and RP among the 3 histological types, whereas a significant correlation existed between histological grading and RE ($P<0{\cdot}02$).**

**Finally, patients with $RE^+$ clinically inflammatory tumours constitute a lower risk group, especially when they are free of metastases at the time of diagnosis. The presence of RE therefore seems to indicate, as in the operable tumour group, a favourable prognosis.**

THE clinically inflammatory tumours are considered to be very severe carcinomas, reflecting serious imbalance in the tumour–host relationship (Denoix, 1970). They are of unfavourable prognosis and are characterized by a particularly low rate of survival at 5 years (~15%) (Fletcher & Montague, 1975; Lacour & Hourtoule, 1967). These tumours very seldom show any of the microscopic features typical of clinically characterized "inflammation" (Haagensen, 1971).

In the present study, we have simultaneously determined oestrogen receptors (RE) and progesterone receptors (RP) in inflammatory tumours of the human breast, and have compared these results with those obtained in primary operable carcinomas. The prognostic value of RE has been studied as well, since the presence of RE should be a favourable prognostic factor, independently of other known factors in primary operable carcinomas (Knight *et al.*, 1977; Fletcher *et al.*, 1978; Cooke *et al.*, 1979).

### MATERIALS AND METHODS

*Patients.*—The present series includes 59 female patients with clinically inflammatory tumours of the breast. Diagnosis and treatment were performed at the Institut Gustave-Roussy between March 1975 and December 1977. Metastatic work-up included chest X-rays, mammograms, X-rays of the pelvis, and bone or liver scans. Inflammatory tumours are defined as tumours associated with local inflammatory symptoms (cutaneous oedema, skin redness and heat). These signs are either localized in a part of or are in the whole breast. The rapidly growing tumours (identified by careful history taking) were not included in this study.

We have compared the 59 measurements to those of 496 primary operable cases; *i.e.* $T_1$, $T_2$ or $T_3 < 7$ cm; $N_0$, $N_1$; $M_0$ according to TNM classification.

*Systemic treatment for patients with inflammatory tumours.*—The gravity of this tumour type is such that systemic treatment must be given priority. This treatment combined chemotherapy, radiotherapy and hormone therapy:

Chemotherapy included several kinds of drugs: either Adriamycin (40 mg/m$^2$ i.v.) + Oncovin (1 mg i.v.) + Methotrexate (8 mg/24 h s.c.) or Oncovin (1 mg i.v.) + 5FU (300 mg/m$^2$ i.v.) + Cyclophosphamide (250 mg/m$^2$ i.v.) and was administered in 18–24 monthly cycles.

Radiotherapy was given in two steps: a first step between the 3rd and the 4th cycle (45 Gy to the breast, chest wall and regional lymph nodes over $4\frac{1}{2}$ weeks) and a second step between the 4th and 5th cycle (20 Gy to the breast over 2 weeks).

Hormone therapy was adapted to the hormonal status of the patient: castration by pelvic irradiation in the premenopausal women with lymph-node involvement and Tamoxifen in the others.

*Receptor assay.*—Immediately after large biopsy under local anaesthesia, tumour specimens (100–200 mg) were transported to the laboratory on ice within a few minutes of operation. Malignancy was verified by histological examination by the pathologist. The samples were crushed at 0°C in 3 volumes of buffer (0·01M Tris-HCl, 0·012M dithiotreitol, 10% glycerol, pH 7·4) with an ultraturrax (3–4 five-second bursts with 15-second cooling intervals) and homogenized in a Teflon-glass homogenizer.

The homogenate was centrifuged for 1 h at 105,000 *g* in the 50 Ti rotor of a Beckman L2 centrifuge. The supernatant (= cytosol) was used to determine RE and RP with a single-saturating-dose method previously described (May–Levin *et al.*, 1977). Tumours with levels of specific-binding sites greater than 100 fmol/g tissue were RE+, RP+. This cut-off was arbitrarily chosen, and corresponds to the limit of sensitivity of our technique (Raynaud *et al.*, 1977); it is comparable to those chosen by several authors (Horwitz *et al.*, 1975; Wittliff *et al.*, 1976). However, for positive results near the cut-off point (3 results between 100 and 200 fmol/g tissue) we considered the tumoral cell density as determined by the pathologist; where cell density was high, results were considered as negative, where it was low, results were positive.

*Miscellaneous.*—We have also determined:

(a) Histological types, which were divided into 3 classes (Scarff & Torloni, 1968; Contesso *et al.*, 1977): "well-differentiated" (tubular or papillary); "pleomorphic" (infiltrating ductal carcinoma with 2 components mixed: tubular and trabecular); "atypical" (infiltrating ductal carcinoma without tubular or papillary components).

Our series had only one medullary adenocarcinoma with lymphoplasmocytic stroma.

(b) Histological grading according to WHO and Scarff and Bloom (Bloom, 1950; Bloom & Richardson, 1957) taking into consideration: the degree of differentiation; nuclear pleomorphism; mitotic activity.

(c) Tumour cell density (see above).

Labelled steroids (oestradiol for RE, R 5020 for RP) were purchased from New England Nuclear Corp. (6072 Dreieich, W. Germany).

Statistical significance between groups was determined by the $\chi^2$ test and adjustment test of Boyd & Doll (1954). Disease-free survival rates were calculated by the Kaplan–Meier method (1958) and curves compared by the log-rank test (Peto *et al.*, 1976, 1977).

### RESULTS

### *Histological characteristics and hormone receptors*

Pre- and post-menopausal cases had the same distribution of all histological characteristics, allowing us to combine the 2 populations for our study.

There was a significant difference ($P < 0{\cdot}001$) between operable and inflammatory tumours in terms of histological type (Table I). Operable tumours were more often pleomorphic, inflammatory tumours more often atypical. On the other hand, no significant difference was found in the distribution of RE among the 3 histological types, either for operable tumours or for inflammatory ones (Table II).

There was a significant difference

TABLE I.—*Histological comparison between operable and inflammatory tumours*

| Histological types | Well-differentiated | Pleomorphic* | Atypical* | Other forms |
|---|---|---|---|---|
| Operable | 23 (5%) | 297 (61%) | 106 (22%) | 60 (12%) |
| Inflammatory | 4 (7%) | 17 (31%) | 34 (62%) | 0 |

$P < 0{\cdot}001$.
Histological typing was not available in 10 operable tumours and 4 inflammatory tumours.
* See Material and Methods.

TABLE II.—*Relationship between histological type and oestrogen receptors in operable and inflammatory tumours* ($RE^- < 100$ *fmol/g tissue*)

| Histological types | | Well-differentiated | Pleomorphic* | Atypical* | |
|---|---|---|---|---|---|
| Operable tumours | $RE^-$ | 8 (35%) | 129 (43%) | 57 (54%) | NS |
| | $RE^+$ | 15 (65%) | 168 (57%) | 49 (46%) | |
| Inflammatory tumours | $RE^-$ | 1 (25%) | 9 (53%) | 18 (53%) | NS |
| | $RE^+$ | 3 (75%) | 8 (47%) | 16 (47%) | |

$RE^+$, $RE^-$ = tumours with and without oestrogen receptors.
$RP^+$, $RP^-$, tumours with and without progesterone receptors.

TABLE III.—*Comparison between operable and inflammatory tumours according to histological grade* (*Scarff–Bloom*)

| Histological grade | I | II | III |
|---|---|---|---|
| Tumours | | | |
| Operable | 85 (17%) | 227 (47%) | 175 (36%) |
| Inflammatory | 0 | 17 (30%) | 40 (70%) |

$P < 0{\cdot}001$.
Histological grading was not available in 7 operable tumours and 2 inflammatory tumours.

($P < 0{\cdot}001$) between the 2 categories of tumours in terms of histological grading (Table III).

A significant correlation was found ($P < 0{\cdot}02$) between histological grading and RE for inflammatory tumours only, as shown in Table IV. Grade III are more frequently $RE^-$ and Grade II more frequently $RE^+$; there was no tumour in Grade I.

The results for RP paralleled those of RE, but the difference was not statistically significant.

*Types of tumours and hormone receptors*

There was a statistically significant difference ($P < 0{\cdot}02$) in the distribution of RE and RP between operable and inflammatory tumours (Table V). In the inflammatory tumours the proportion of ($RE^-$, $RP^-$) cases was higher (48%) than in the operable group (28%).

This difference applied to both pre- and postmenopausal women, as shown in Table VI. Significance was obtained in an adjustment test (Boyd & Doll, 1954).

*Prognostic value of hormone receptors*

We have tried to determine whether RE status influenced the evolution of the disease. This has been done by studying the disease-free curves of $RE^+$ and $RE^-$ patients (Kaplan & Meier, 1958). For inflammatory tumours, Fig. 1 shows that the number of cases presenting with metastases at diagnosis is the same for $RE^+$ tumours (11/28 patients) as for $RE^-$ tumours (10/30 patients). The slopes of

TABLE IV.—*Relationship between histological grading and oestrogen receptors in operable and inflammatory tumours*

| Histological grading | | I | II | III | |
|---|---|---|---|---|---|
| Operable tumours | $RE^-$ | 35 (41%) | 107 (47%) | 87 (50%) | NS |
| | $RE^+$ | 50 (59%) | 122 (53%) | 88 (50%) | |
| Inflammatory tumours | $RE^-$ | 0 | 5 (29%) | 25 (62%) | $P < 0{\cdot}02$ |
| | $RE^+$ | 0 | 12 (71%) | 15 (38%) | |

TABLE V.—*RE and RP in human mammary carcinomas*

| Receptors status | Tumours | |
|---|---|---|
| | Operable | Inflammatory |
| RE⁻, RP⁻ | 28% (140) | 48% (28) |
| RE⁺, RP⁻ | 18% (91) | 19% (11) |
| RE⁻, RP⁺ | 15% (76) | 5% (3) |
| RE⁺ RP⁺ | 38% (189) | 28% (17) |

$P < 0{\cdot}02$.

the curves, however, show more favourable evolution for patients with RE⁺ tumours ($P < 0{\cdot}01$). If we exclude patients with metastases at diagnosis, we obtain a more significant difference (log-rank, ($P < 0{\cdot}001$) between the disease-free curves of patients with RE⁺ and RE⁻ tumours (Fig. 2). Concerning the operable tumours, the prognostic value of RE is under study; we operate very severe patient selection, and we consider that a follow-up of only 36 months is insufficient for a population with a good prognosis (80% at 5 years).

FIG. 2.—RE and recurrence in patients free of metastasis at the time of diagnosis of inflammatory tumours.
RE⁻--- (n = 20).
RE⁺—— (n = 17).
$P < 0{\cdot}001$.

FIG. 1.—RE and recurrence in all patients with clinically inflammatory tumours.
RE⁻--- (n = 30) (1 patient lost to view).
RE⁺—— (n = 28).
$P < 0{\cdot}01$.

### DISCUSSION

Until now, inflammatory tumours of the breast have been categorized on the basis of clinical criteria alone. The gravity of this tumour type is such that systemic treatment must be given priority (see Materials and Methods). This study has

TABLE VI.—*RE and RP in human mammary carcinomas according to hormonal status*

| Hormonal status | Tumours | Receptors | | | |
|---|---|---|---|---|---|
| | | RE⁻, RP⁻ | RE⁺, RP⁻ | RE⁻, RP⁺ | RE⁺, RP⁺ |
| Premenopausal | Operable | 30% (66) | 10% (23) | 21% (48) | 39% (87) |
| | Inflammatory | 50% (15) | 13% (4) | 3% (1) | 33% (10) |
| Postmenopausal | Operable | 27% (74) | 25% (68) | 10% (28) | 38% (102) |
| | Inflammatory | 45% (13) | 24% (7) | 7% (2) | 24% (7) |

$P < 0{\cdot}02$ with the adjustment test of Boyd & Doll (1954).
The number of patients is indicated in brackets.

confirmed the high frequency of distant metastases at diagnosis (21/59, or 36%), but until now, it has not been possible either to identify the mechanisms responsible for the clinical identity of these tumours, or to determine the relevance of certain aetiological factors (age, family history, endocrine status, *etc.*).

Histological results confirmed that this is a separate disease entity, 62% of the tumours atypical, which agrees with other authors (*e.g.* Haagensen, 1971) who found that the atypical type accounted for 80% of 59 cases. As opposed to our results, Martin *et al.* (1978) found a relationship between histological type and RE distribution, on the basis of classification into 2 categories: well-differentiated and pleomorphic with more than 50% differentiation, and atypical and pleomorphic with less than 50% differentiation.

As for the histological grading of Scarff and Bloom, as has already been observed (Sarrazin *et al.*, 1978) we find these inflammatory tumours to be highly malignant with 70% being classified as Grade III, the most unfavourable group. While a significant relationship between histological grading and RE distribution could be shown only for the inflammatory tumours, such a relationship (though not statistically significant) was also seen for the operable tumours. Our results are identical to those reported by other groups (Maynard *et al.*, 1978; Furmanski *et al.*, 1980; King, 1980; McCarty *et al.*, 1980; Millis, 1980). Taken together, these results confirm that RE and RP receptors represent an aspect of cell differentiation of tumours. Furthermore, other authors (Meyer *et al.*, 1977; Silvestrini *et al.*, 1979) have shown that tumours with a high growth rate and labelling index are most frequently without receptors.

The fact that inflammatory tumours show a higher proportion of $RE^-$, $RP^-$ cases than the operable tumours is a further argument for distinguishing between the 2 tumour types (McGuire *et al.*, 1975, 1977; May-Levin *et al.*, 1977). Our results thus indicate that, amidst this population of inflammatory tumours with a generally poor prognosis, there is a histo-biological subgroup with a still worse prognosis.

The clinical progression seems to confirm these observations. The prognostic value of RE for the inflammatory tumours is in keeping with results published for the operable tumours (Knight *et al.*, 1977; Fletcher *et al.*, 1978; Cooke *et al.*, 1979; Furmanski *et al.*, 1980; Osborne *et al.*, 1980) even if sometimes (Blamey *et al.*, 1980) the prognostic value is limited to the $N^+$ tumours. Only Hilf *et al.* (1980*a,b*) found no favourable prognostic index for the operable $RE^+$ tumours. Nevertheless, we must point out that patients in our study were all subjected to the same systemic treatment; it is thus possible that the efficiency of the hormonal treatment is at least partially responsible for the favourable evolution of $RE^+$ tumours, which should be hormone sensitive.

Finally, we consider it interesting that the frequency of $RE^+$ tumours is the same in the pre-menopausal (46%) and the post-menopausal (48%) groups. A study carried out in parallel with ours at the Institute Gustave-Roussy (Rouësse *et al.*, 1979) for comparison of current treatment modalities (chemotherapy and hormone therapy) for inflammatory tumours with the previous surgical approaches, has shown that only pre-menopausal women benefit from this approach (both in terms of survival rate and duration of remission). However, RE assay could not be carried out in all patients, so that no correlation could here be drawn.

The authors acknowledge the technical assistance of Mireille Lemaout.

### REFERENCES

BLAMEY, R. W., BISHOP, H. M., BLAKE, J. R. S. & 5 others (1980) Relationship between primary breast tumor receptor status and patient survival *Cancer*, **46**, 2765.

BLOOM, H. J. G. (1950) Prognosis in carcinoma of the breast. *Br. J. Cancer*, **4**, 259.

BLOOM, H. J. G. & RICHARDSON, W. W. (1957) Histological grading and prognosis in breast cancer. A study of 1409 cases of which 359 have been followed 15 years. *Br. J. Cancer*, **11**, 359.

BOYD, J. T. & DOLL, R. (1954) Gastro-intestinal cancer and the use of liquid paraffin. *Br. J. Cancer*, **8**, 231.

CONTESSO, G., ROUESSE, J., PETIT, J. Y. & MOURIESSE, H. (1977) Les facteurs anatomopathologiques du pronostic des cancers du sein. *Bull. Cancer*, **64**, 525.

COOKE, J., GEORGE, O., SHIELDS, R., MAYNARD, P. & GRIFFITHS, K. (1979) Estrogen receptors and prognosis in early breast cancer. *Lancet*, i, 995.

DENOIX, P. (1970) Treatment of malignant breast tumors. *Recent Results in Cancer Research*, **31**.

FLETCHER, W. S. & MONTAGUE, F. O. (1975) Radical irradiation of advanced breast cancer. *Am. J. Roentgenol. Radium Ther. Nucl. Med.*, **3**, 573.

FLETCHER, W. S., LEUNG, B. S. & DAVENPORT, C. E. (1978) The prognostic significance of estrogen receptors in human breast cancer. *Am. J. Surgery*, **135**, 372.

FURMANSKI, P., SAUNDERS, D. E., BROOKS, S. C. & RICH, M. A. (1980) The prognostic value of estrogen receptor determinations in patients with primary breast cancer. *Cancer*, **46**, 2794.

HAAGENSEN, C. D. (1971) Inflammatory Carcinoma. In *Diseases of the Breast*. Philadelphia: Saunders. p. 576.

HILF, R., FELDSTEIN, M. L., GIBSON, S. L. & SAVLOV, E. O. (1980*a*) The relative importance of estrogen receptor analysis as a prognostic factor for recurrence of response to chemotherapy in women with breast cancer. *Cancer*, **45**, 1993.

HILF, R., FELDSTEIN, M. L., SAVLOV, E. O., GIBSON, S. L. & SENECA, B. (1980*b*) The lack of relationship between estrogen receptor status and response to chemotherapy. *Cancer*, **46**, 2797.

HORWITZ, K. B., MCGUIRE, W. L., PEARSON, O. H. & SEGALOFF, A. (1975) Predicting response to endocrine therapy in human breast cancer: A hypothesis. *Science*, **189**, 726.

KAPLAN, E. L. & MEIER, P. (1958) Nonparametric estimation from incomplete observations. *J. Am. Statist. Ass.*, **53**, 457.

KING, R. J. B. (1980) Analysis of estradiol and progesterone receptors in early and advanced breast tumors. *Cancer*, **46**, 2818.

KNIGHT, W. A., LIVINGSTON, R. B., GREGORY, E. J. & MCGUIRE, W. L. (1977) Estrogen receptor as an independent prognosis factor for early recurrence in breast cancer. *Cancer Res.*, **37**, 4669.

LACOUR, J. & HOURTOULE, F. G. (1967) La place de la chirurgie dans le traitement des formes évolutives du cancer du sein. *Mém. Acad. Chirurgie*. p. 635.

MARTIN, P. M., JACQUEMIER, J., ROLLAND, P. H., ROLLAND, A. M. & TOGA, M. (1978) Correlations entre les récepteurs hormonaux stéroidïens et l'anatomopathologie des tumeurs mammaires humaines. *Bull. Cancer*, **65**, 383.

MAY-LEVIN, F., GUERINOT, F., CONTESSO, G., DELARUE, J. C. & BOHUON, C. (1977) Etude des récepteurs cytosoliques estrogènes et progestogènes dans les carcinomes mammaires. *Int. J. Cancer*, **19**, 789.

MAYNARD, P. V., DAVIES, C. J., BLAMEY, R. W., ELSTON, C. W., JOHNSON, J. & GRIFFITHS, K. (1978) Relationship between oestrogen-receptor content and histological grade in human primary breast tumour. *Br. J. Cancer*, **38**, 745.

MCCARTY, K. S. JR, BARTON, T. K., FETTER, B. F. & 6 others (1980) Correlations of estrogen and progesterone receptors with histologic differentiation in mammary carcinoma. *Cancer*, **46**, 2851.

MCGUIRE, W. L., VOLLMER, E. P. & CARBONE, P. P. (Eds) (1975) *Estrogen Receptors in Human Breast Cancer*. N.Y.: Raven Press.

MCGUIRE, W. L., RAYNAUD, J. P. & BAULIEU, E. E. (Eds) (1977) *Progesterone Receptors in Normal and Neoplastic Tissues*. N.Y.: Raven Press.

MEYER, J. S., RAO, B. R., STEVENS, S. C. & WHITE, W. L. (1977) Low incidence of estrogen receptor in breast carcinomas with rapid rates of cellular replication. *Cancer*, **40**, 2290.

MILLIS, R. R. (1980) Correlation of hormone receptors with pathological features in human breast cancer. *Cancer*, **46**, 2869.

OSBORNE, C. K., YOCHMOWITZ, M. G., KNIGHT, W. A. & MCGUIRE, W. L. (1980) The value of estrogen and progesterone receptors in the treatment of breast cancer. *Cancer*, **46**, 2884.

PETO, R., PIKE, M. C., ARMITAGE, P. & 7 others (1976, 1977) Design and analysis of randomized clinical trials requiring prolonged observation of each patient. I. *Br. J. Cancer*, **34**, 585. II, *Br. J. Cancer*, **35**, 1.

RAYNAUD, J. P., BOUTON, M. M., PHILIBERT, D., DELARUE, J. C., GUERINOT, F. & BOHUON, C. (1977) Progesterone and estradiol binding sites in human breast carcinoma. In *Research in Steroids*, **7**, 281.

ROUESSE, J., SARRAZIN, D., MAY-LEVIN, F., AMIEL, J-L., BRULE, G. & MOURIESSE, H. (1979) Effect of chemotherapy pre and post local irradiation in the treatment of inflammatory breast cancer. In *2nd Breast Cancer Working Conference*. Copenhagen: E.O.R.T.C.

SARRAZIN, D., ROUESSE, J., ARRIAGADA, R., MAY-LEVIN, F., PETIT, J. Y. & CONTESSO, G. (1978) Les cancers du sein en "poussée evolutive". *Rev. Prat.*, **28**, 999.

SCARFF, R. W. & TORLONI, H. (1968) *International Histological Classification of Tumors*, 2. Geneva: World Health Organization. 204.

SILVESTRINI, R., DAIDONE, M. G. & DI FRONZO, G. (1979) Relationship between proliferative activity and estrogen receptors in breast cancer. *Cancer*, **44**, 665.

WITTLIFF, J. L., BEATTY, B. W., SAVLOV, E. D., PATTERSON, W. B. & COOPER, R. A. (1976) Estrogen receptors and hormone dependency in human breast cancer. In *Recent Results in Cancer Res.*, **57**, 59.